# Special Issue “Ion Channels as a Therapeutic Target: Drug Design and Pharmacological Investigation 2.0

**DOI:** 10.3390/ijms27010395

**Published:** 2025-12-30

**Authors:** Gabriella Guerrini, Maria Paola Giovannoni

**Affiliations:** Neurofarba, Pharmaceutical and Nutraceutical Section, University of Florence, Via Ugo Schiff 6, 50019 Sesto Fiorentino, Italy; mariapaola.giovannoni@unifi.it

**Editorial on the Research Topic**

**Ion Channels as Therapeutic Target: Drug Design and Pharmacological Investigation 2.0**

This Special Issue highlights recent advances in ion channel research with a set of articles that enriches this topic, primarily through new research in medicinal chemistry and pharmacology aimed at identifying potential new drugs, including those derived from natural products. The involvement and contribution of sodium, potassium, and TRP channels in neuronal, anesthetic, hippocampal, and wound-healing activities are discussed in the papers herein, as was the importance of water and protons to the opening/closing of the ion channel.

Moreira-Junior et al. (contribution 1) present a study on anethole (ANE), a phenolic compound synthesized by many aromatic plants, and its effects on voltage-gated Na^+^ channels (VGSCs). While the interaction of ANE with various ion channels (TRPA1, TRP, ad Ca^2+^) and its pharmacological properties (anti-inflammatory, anticancer, and neuroprotective) are well-documented, studies regarding its direct interaction with VGSCs are scarce, and the authors aim was to fill this gap. More specifically, the authors studied the effects of ANE on sensory neurons in rats to evaluate the channels in their native physiological environment using patch-clamp recordings. Their research shows that ANE is efficient when it comes to blocking VGSCs, with different affinities in the resting or inactivated states of the channels, and the channel block is fully reversible and concentration-dependent. Since VGS channels are essential for the generation and propagation of action potentials and are associated with neuronal excitability, blocking them with ANE in the inactivated state is advantageous in neurological diseases such as depression, stress, epilepsy, and absence seizures [1,2]. Moreover, the ability of ANE to modulate cell excitability by blocking the sodium current (I_Na+_/VGSCs), via a mechanism similar to that of lidocaine, suggests its potential use as a local anesthetic as well as the development of analogs compounds for pain treatment.

The anesthetic effect of switchable drugs acting as voltage-gated sodium (Na_v_) channel blockers was studied by Noev et al. (contribution 2). The authors had previously reported on the synthesis of ethercaine hydrochloride ([4-(2-(N-Morpholino)-Ethoxy)-Azobenzene Hydrochloride] [3,4]), which shows local anesthetic properties if irradiated with blue or white light, while after irradiation with UV light (360 or 395 nm), the biological activity disappeared. They now report on the synthesis of new derivatives of ethercaine, bearing an alkoxyl moiety or fluorine atom in different positions of the azobenzene group, replacing the nitrogen-containing fragment present in ethercaine (with a morpholine, piperazine, and piperidine moiety) or introducing a bioisosteric substitution of a phenyl ring with a heterocycle ring, to obtain derivatives with improved water solubility and with a light-controlled local anesthetic profile. All compounds were analyzed in vitro to evaluate the cytotoxic effect on human dermal fibroblasts (DF2); the anesthetic activity was assessed in vivo in a model of surface anesthesia in both darkness and under UV light irradiation; the Z-E isomerization half-life and the stability of the respective geometric isomers suggests that the introduction of fluorine atoms onto the ethercaine scaffold produces photoswitchable derivatives, as does introducing of thiazole heterocycle, which also shows good water solubility. Despite photopharmacological approaches having already been applied to drugs acting on the Na_v_ channel and showing different pharmacological profiles, such as anesthetics or antiarrhythmics and on other ion channels [5], information related to structure–activity relationships (SARs) remains scarce; therefore, this study could contribute to greater comprehension of this very intriguing field.

Eisfeld et al. (contribution 3) reports an investigation about the blocking of the Na_v_1.7 sodium channel by the alkaloid plant Harmaline, used in traditional medicine in North Africa, and produced by the plant *Peganum harmala*. Harmaline and other alkaloids present in the African plant belong to the β-carboline chemical class, and their traditional uses include the treatment of asthma, hypertension, diabetes, and chronic pain [6], even though elevated dosages produce neurological effects such as hallucinations, agitation, tremors. This could reflect the broad impact of harmaline on various biological targets, including the Ca^2+^ voltage-dependent channels, several G-protein-coupled receptors, and monoamine oxidase (MAO) [4]. On the other hand, since the use of harmaline in traditional medicine is related to pain relief, the authors aimed to evaluate the mechanisms and actions of this natural compound in the nociceptive system, with a particular focus on the implicated Na_v_1.7 channels Additional emphasis was placed on two rare forms of pain perception: inherited erythromelalgia and paroxysmal extreme pain disorder. The authors, through an innovative patch-clamp system, study the hNa_v_1.7 channels expressed in CHO cells, and their results indicate that harmaline blocks the voltage-gated sodium channel in a dose-dependent manner (IC_50_ 35.5 μM), with an effect that is not channel state-dependent. Moreover, they also describe a peripheral target of harmaline and its ability in pain relief that had not been reported on to date. However, despite these intriguing results, the authors remain skeptic about the use of all ‘components’ of *P. harmala*, due to its manifest neurotoxicity.

The effects on potassium channels of the newly identified conopeptide Cs68 (kO-SrVIA), from the venom of the vermivorous *Conus spurius* species native to the Gulf of Mexico, were analyzed by Martínez-Hernández et al. (contribution 4). The potassium channels studied by these authors belong to the EAG category (ether-à-go-go or KCNH), a subfamily of voltage-gated potassium channels, whose activation drives membrane potential. As voltage-dependent channels, when the membrane potential changes, they switch between open and closed conformations, thus influencing the passage of potassium ions. The conotoxins are conopeptides generally formed by 12–40 amino acids and exert a broad and potent activity toward various biological targets: ion voltage-gated channels, nAchR, NMDAR, 5HT_3_R, and G-protein-coupled receptors. To date, only thirteen conopeptides are known to interact with potassium channels, in particular with K_v_1.6, so it could be very interesting if the newly identified conopeptide Cs68 is able to interact with K_v_1.10 and K_v_1.11, in addition to K_v_1.6. The new peptide has 31 residues, three disulfide bridges, and a free C-terminus; in accordance with the conotoxin nomenclature, it was named kO-SrVIA, as it belongs to the O1 superfamily and shares about 70% sequence similarity with the δ-like-AtVIA family [7].

With the results of an electrophysiological assay, the authors demonstrate that the new conopeptide inhibits all three voltage-gated potassium channels (K_v_1.6, K_v_10.1, and K_v_11.1), with IC50 values of 3.6, 1.88, and 2.44 μM, respectively. In their opinion, the new conopeptide kO-SrVIA could be a promising molecule to study, especially since blocking the K_v_10.1 channel induces a reduction in cell proliferation in different cancer lines [8]. On the other hand, the slight difference affinity between the K_v_10.1 and K_v_11.1 channels observed for kO-SrVIA will require further efforts in terms of designing conopeptide kO-SrVIA analogs to achieve better selectivity towards K_v_10.1.

The study by Phelan et al. (contribution 5) focuses on Canonical Transient Receptor Potential Channel 3 (TRPC3), the most abundant receptor of TRPC in the hippocampus. Although its role in this brain area has not been thoroughly investigated, it could be involved in the hyperexcitability of pyramidal neurons and seizures. TRPCs are a subfamily of TRP cation channels and, until now, seven mammalian members are described as TRPC1-7, mainly activated by various G-protein-coupled receptors or directly by diacylglycerol (DAG), and all are permeable to mono- or divalent cations (Na, K, or Ca). TRPC3s are also activated by tyrosine kinase, oxidative stress, and mechanical stimuli, making this target very intriguing for drug design. The authors investigate the role of TRPC3 channels in hippocampal pyramidal neurons and their involvement in epileptiform discharges, as well as the electrophysiological properties of these neurons. Surprisingly, the results of this study indicate that TRPC3 channels contribute little to the excitability of pyramidal neurons in mature animals; this contradicts previous research performed by the same authors on young mice [9], as well as evidence that supports genetic ablation of TRPC3 expression results in a reduction in spontaneous burst firing and of the status epilepticus induced by neuronal cell death in hippocampal neurons. The complexity of this neuronal system, however, is far too great to be so simply resolved, and further studies are needed to explore whether TRPC3 channel blockers could have therapeutic potential.

In the review of Grigore et al. (contribution 6), the role of Transient Receptor Potential (TRP) in wound-healing was examined. The authors scanned the literature (tag: ion channel and wound-healing) from 1 January 2023, to the present and excluded all papers that did not present the involvement of ion channels in the pharmacological mechanism of wound-healing. In particular, they focused their attention on 46 papers that report in vitro (38) and in vivo experiments on rats and mice (8), addressing the in vivo mechanism of wound-healing. Their analysis detailed the tissue repair phases and the various ion channels involved in epidermal re-epithelialization. Damage to the skin and vascular endothelium led to hemostasis, followed by the formation and subsequent degradation of the fibrin clot, and the inflammatory phase typically lasts 3 to 5 days. The formation of granulation tissue, as well as epithelization and the proliferation of fibroblasts, the reorganization of the extracellular matrix, and the development of blood vessels, represent the final steps in wound-healing, culminating in the formation of a scar [10]. In this mechanism, several types of ions (Ca^2+^, Na^+^, and K^+^), which are also responsible for the Transepithelial potential (TEP) and the Epithelial electric field (EF), are involved. The electric fields, currents, and endogenous electrical signals that appear immediately when a skin lesion occurs seem to be the first stimulus for tissue repair through cell migration. The main ion channels involved in the tissue repair processes belong to TRP channels such as TRPC (Canonical Transient Receptor Potential Channel, TRPC1-TRPC6) expressed in keratinocytes; TRPV (Transient Receptor Potential Channel Vanilloid) expressed in neutrophils (TRPV1), mast cells, macrophages, natural killer cells (TRPV2), hair follicles, basal layer of epidermis (TRPV3), skin cells (TRPV4); and TRPM (Transient Receptor Potential Channel Melastatin) expressed in endothelial and vascular smooth muscle cells (TRPM1, TRPM5, and TRPM8) [11]. The authors explain in detail each channel’s positive or negative influence on wound-healing in murine models, but they are conscious that this contribution is likely not sufficient to elucidate the mechanism of wound-healing in humans, given that it lacks the relevant in vivo studies.

Finally, the review by Michael E. Green and Alisher M. Kariev (contribution 7) discusses the role of water and protons in the gating of ion channels (K^+^, Na_v_, and H_v_1). The opening of voltage-gated ion channels, inducing a change in membrane potential and the subsequent physiological effects, is followed by conduction closure via a channel inactivated state, then the cycle of opening and conduction repeats. In some cases, protons may be partially responsible for gating. Since 1989, the importance of water molecules and the ‘mode’ in which these molecules are gathered in the channel—e.g., blocked to form a frozen configuration or mobile—has been increasingly acknowledged. On the other hand, even if the descriptions of a ‘frozen configuration of water’ cannot be considered correct, the arrangement of the water molecules near/in proximity to/along the gate is verisimilar; in this context, even protons may play a crucial role in the opening/closing of the gate channel, even inducing a rearrangement/movement of protein side chains. As an alternative to the standard gating model in which segment S4 (of the ion channel) moves to mechanically close the pore [12], the authors try to understand the importance of water and proton movement in response to a variation in voltage in the gate mechanism. The membrane surface and the water molecules in the membrane surface and/or in the gate region, as well as fluctuations in the ‘amount of water’ in the channel, seem to be important to ‘gating questions’, even if research in this area is still ongoing. Another aspect the authors pointed out is the lack of valid Molecular Dynamic (MD) models, which do consider water molecules, but also the charge transfer or bond-breaking [13]. The issue is very complex, and the authors contribute to making it more accessible to the community of interested experts.
